# Transit time flow measurement of coronary bypass grafts before and after protamine administration

**DOI:** 10.1186/s13019-021-01575-y

**Published:** 2021-07-09

**Authors:** Dror B. Leviner, Miriam von Mücke Similon, Carlo Maria Rosati, Andrea Amabile, Daniel J. F. M. Thuijs, Gabriele Di Giammarco, Daniel Wendt, Gregory D. Trachiotis, Teresa M. Kieser, A. Pieter Kappetein, Stuart J. Head, David P. Taggart, John D. Puskas

**Affiliations:** 1 Department of cardiac surgery, Carmel Medical Center, 3436212 Haifa, Israel; 2 Sackler School of Medicine New York State/American Program, Tel Aviv University, Tel Aviv, Israel; 3 Department of Cardiovascular Surgery, Mount Sinai Morningside, New York, NY USA; 4 Division of Cardiac Surgery, Department of Surgery, Yale University School of Medicine, New Haven, CT USA; 5 Department of Cardiothoracic Surgery, Erasmus MC, University Medical Center, Rotterdam, Netherlands; 6 Clinica Cardiochirurgica, Università “G.D’Annunzio”, Chieti, Italy; 7 Department of Thoracic and Cardiovascular Surgery, West German Heart Center, Essen, Germany; 8 Heart center, Veterans Affairs Medical Center, Washington, DC USA; 9 LIBIN Cardiovascular Institute of Alberta, Calgary, AB Canada; 10 Department of Cardiovascular Surgery, University of Oxford, Oxford, UK

**Keywords:** Coronary artery bypass grafting, Quality control, Intraoperative graft flow measurement

## Abstract

**Background:**

Intraoperative graft assessment with tools like Transit Time Flow Measurement (TTFM) is imperative for quality control in coronary surgery. We investigated the variation of TTFM parameters before and after protamine administration to identify new benchmark parameters for graft quality assessment.

**Methods:**

The database of the REQUEST (“REgistry for QUality AssESsmenT with Ultrasound Imaging and TTFM in Cardiac Bypass Surgery”) study was retrospectively reviewed. A per graft analysis was performed. Only single grafts (i.e., no sequential nor composite grafts) where both pre- and post-protamine TTFM values were recorded with an acoustical coupling index > 30% were included. Grafts with incomplete data and mixed grafts (arterio-venous) were excluded. A second analysis was performed including single grafts only in the same MAP range pre- and post- protamine administration.

**Results:**

After adjusting for MAP, we found a small increase in MGF (29 mL/min to 30 mL/min, *p* = 0.009) and decrease in PI (2.3 to 2.2, *p* <  0.001) were observed after the administration of protamine. These changes were especially notable for venous conduits and for CABG procedures performed on-pump.

**Conclusion:**

The small changes in TTFM parameters observed before and after protamine administration seem to be clinically irrelevant, despite being statistically significant in aggregate. Our data do not support a need to perform TTFM measurements both before and after protamine administration. A single TTFM measurement taken either before or after protamine may suffice to achieve reliable data on each graft’s performance. Depending on the specific clinical situation and intraoperative changes, more measurements may be informative.

**Trial registration:**

Clinical Trials Number: NCT02385344, registered February 17th, 2015.

**Supplementary Information:**

The online version contains supplementary material available at 10.1186/s13019-021-01575-y.

## Background

Intra-operative assessment of graft patency is crucial to perform high-quality coronary artery bypass graft (CABG) procedures. Occlusion rates as high as 20.0 and 8.0% have been reported at 1-year follow-up for venous and arterial conduits, respectively [1, 2]. Graft failure within the first year of surgery may be in part attributed to technical errors [3, 4], which can lead to graft kinking, overstretching, lumen occlusion and anastomotic stenosis, thus resulting in incomplete revascularization [5].

Transit-Time Flow Measurement (TTFM) is an intra-operative tool to assess the patency and the quality of a graft in order to prevent errors from going unnoticed. TTFM relies on specific flow parameters, namely mean graft flow (MGF), pulsatility index (PI), diastolic filling (DF) and backflow (BF). Importantly, no single-parameter measurements have been demonstrated to predict the quality and long-term patency of a graft.

TTFM has been reported to have a 2–4% detection rate of intra-operative errors, and despite the recommendation for its use (class IIa) in the 2018 European guidelines for myocardial revascularization, its adoption has not yet become widespread [6, 7] with an estimate of a global average use of TTFM of ~ 30% but with much regional variance [Medistim data]. This can be attributed in part to a lack of clear cut-off values and the varying sensitivity and specificity of each parameter to predict graft patency.

Performing a retrospective review of the large, multicenter REQUEST (REgistry for QUality AssESsmenT with Ultrasound Imaging and TTFM in Cardiac Bypass Surgery) registry [8], we investigated the variation of the most commonly used TTFM parameters before and after the administration of protamine to identify new benchmark parameters for graft quality assessment, with the hope to increase both the practice and caliber of intraoperative quality control. No study has to this day attempted to investigate the change in TTFM parameters before and after the administration of protamine. Additionally, there are no guidelines as to whether TTFM measurements must be performed both before and after protamine administration or if one measurement will suffice.

## Methods

### Study design

The Registry for Quality Assessment with Ultrasound Imaging and TTFM in Cardiac Bypass Surgery (REQUEST) is an international, multicenter, prospective registry that included 1016 patients in seven cardiac surgery centers (four in Europe and three in North America) between April 2015 and December 2017. These patients underwent isolated coronary artery bypass grafting (CABG) with intraoperative assessment of multiple potential surgical sites, including the ascending aorta (for cannulation, cross clamping and proximal anastomoses, if any), coronary targets, conduits and finally proximal and distal anastomoses using high frequency ultrasound (HFUS) and graft assessment using transit time flowmetry (TTFM) with the MiraQ™ or VeriQ C™ devices (Medistim ASA, Oslo, Norway).

The registry was designed to capture information on any changes in the proposed surgical procedure based on HFUS and/or TTFM findings. These results, together with the study protocol, were reported in a previous publication [8].

The original REQUEST study was funded by Medistim, yet this current study received no funding from any source. The principal investigators and authors had complete scientific freedom. The original REQUEST study is registered at ClinicalTrials.gov (NCT02385344).

### Overall patient population

Patients diagnosed with multivessel coronary artery disease and scheduled for isolated CABG were eligible to be included in the REQUEST registry. Patients were excluded from enrolment if undergoing emergency surgery, when concomitant surgical procedures were planned (e.g., valve repair or replacement, surgery for atrial fibrillation, etc.), if the medical history included the presence of a myopathy, or when the patient was known to be suffering from any psychological, developmental, or emotional disorder. From the original REQUEST trial 1016 patients were included out of the total 1046. Out of the 30 patients excluded, 8 were due to screening failure, 11 because surgical team members were not trained according to the REQUEST study protocol, and 11 because TTFM or HFUS images were unavailable for analysis.

The decision between performing the CABG operation with vs. without cardiopulmonary bypass (on-pump vs. off-pump, or ONCAB vs. OPCAB, respectively) was left to the discretion of the operating surgeon.

### Intra-operative graft assessment with transit-time flowmetry

It was highly recommended, but not mandatory, to intra-operatively assess with TTFM each conduit used for CABG. Only TTFM measurements with an acoustic coupling index (ACI) (as a correlate of the quality or reliability of TTFM measurements) above 30% (both before and after the administration of protamine for heparin reversal) were included in the analysis.

The following four TTFM parameters were measured and recorded: mean graft flow (MGF), pulsatility index (PI), diastolic filling (DF) and backflow (BF).

The systemic mean arterial pressure (MAP) at the time of TTFM was recorded and classified in six ranges: < 53 mmHg, 53–63 mmHg, 64–74 mmHg, 75–85 mmHg, 86–96 mmHg, or > 96 mmHg.

### Inclusion and exclusion criteria (Fig. 1)

In the first stage of the analysis, we considered only single grafts (i.e. with only one distal anastomosis and one or no proximal anastomosis) with pre- and post-protamine TTFM performed and pre- and post-protamine ACI both above 30%. There were 702 patients with a total of 1335 grafts that met these requirements. Sequential grafts were excluded because their presence could add confounders to the analysis. Our analysis remained tailored to single grafts only as TTFM parameters before and after protamine were still undefined and unpublished.

**Fig. 1:**
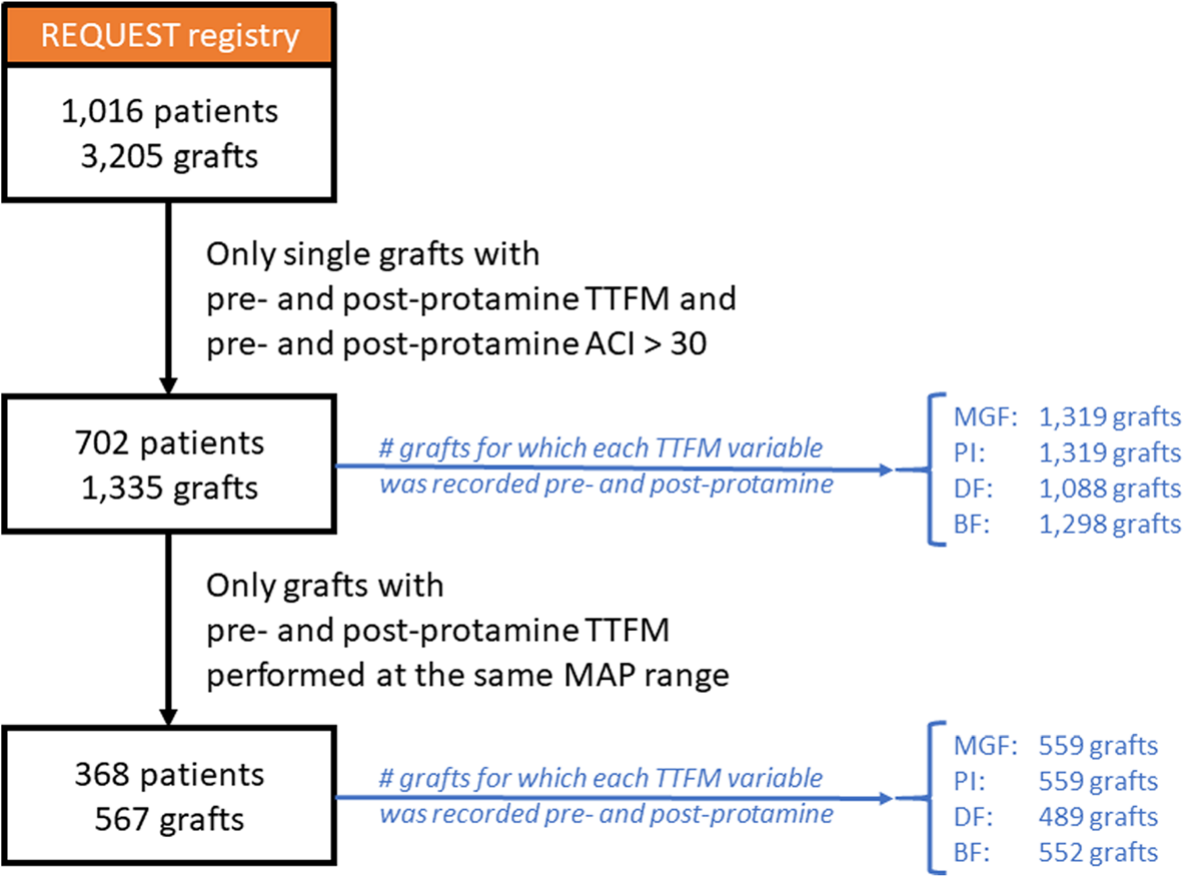
Patient and graft inclusion and exclusion flow chart. ACI = acoustic coupling index; MAP = mean arterial pressure; REQUEST = Registry for Quality Assessment with Ultrasound Imaging and TTFM in Cardiac Bypass Surgery; TTFM = transit-time flowmetry

We then repeated the analysis only for those grafts with the same MAP (i.e., within the same MAP range for each graft) pre- and post-protamine. There were 368 patients with 567 grafts that met these criteria for analysis.

### Statistical analysis

Continuous data were reported as median (25th percentile – 75th percentile, i.e., interquartile range), and categorical data as number (percentage). Comparisons were performed with the chi square, the exact Fisher and the Wilcoxon rank-sum tests, with individual *p*-values < 0.05 deemed statistically significant. Analyses were performed using the open-access software R (https://www.r-project.org/).

## Results

Graft and patient selection as previously described are illustrated in Fig. 1. The baseline characteristics and intra- and post-operative in-hospital variables for the 368 patients with at least a single graft with pre- and post-protamine ACI above 30 and same MAP range pre- and post-protamine are reported in Table 1. Mean age was 67 with a majority (84.5%) of male patients. Slightly more than half of the procedures (56.8%) were performed on-pump. Adverse event rates was extremely low with a mortality of 0.5% and a stroke rate of 1.4%.

**Table 1:** Baseline characteristics and intra- and post-operative in-hospital variables for the 368 patients with at least a single graft with pre- and post-protamine ACI above 30 and same MAP range

	Specifications	*N* = 368 patients
		% (n/N)
**Baseline characteristics**		
Age (years)	Median (range)	67 (36–85)
	≥ 70 years	37.5 (138/368)
Gender, female		15.5 (57/368)
Body mass index (kg/m^2^)	Median (range)	28.0 (15.5–55.0)
Diabetes mellitus		37.5 (106/368)
History of stroke		5.4 (20/368)
History of myocardial infarction		35.3 (130/368)
History of revascularization		
CABG		0.3 (1/368)
PCI		25.8 (95/368)
NYHA classification	I	36.5 (123/337)
	II	44.5 (150/337)
	III	14.8 (50/337)
	IV	4.2 (14/337)
**On- vs. off-pump**		
ONCAB		56.8 (209/368)
OPCAB		43.2 (159/368)
**In-hospital post-operative MACCE**		
Death		0.5 (2/368)
Myocardial infarction		0
Stroke		1.4 (5/368)
Repeat revascularization		0

*CABG* Coronary artery bypass grafting, *MACCE* Major adverse cardiac and cerebrovascular events, *NYHA* New York Heart Association, *ONCAB* On-pump coronary artery bypass, *OPCAB* Off-pump coronary artery bypass, *PCI* Percutaneous coronary intervention

The pre- and post-protamine TTFM variables for the eligible grafts are reported in Table 2. MGF increased post-protamine for venous grafts from 30 to 33 mL/min but remained unchanged for arterial grafts. PI decreased for both venous (2.3 to 2.0) and arterial grafts (2.3 to 2.2).

**Table 2:** Transit-time flowmetry parameters before and after administration of protamine: single grafts with pre- and post-protamine TTFM with ACI > 30 (both pre- and post-protamine)

	N all grafts	N arterial grafts	N venous grafts	All			Arterial grafts			Venous grafts		
				Pre- protamine	Post-protamine	*p* value	Pre-protamine	Post-protamine	*p* value	Pre-protamine	Post-protamine	*p* value
MGF	1319	704	615	29 (17–47)	30 (18–49)	0.02	27 (17–44)	27 (17–45)	0.77	30 (18–52)	33 (20–53)	< 0.001
PI	1319	704	615	2.3 (1.7–3.2)	2.1 (1.6–2.8)	< 0.001	2.3 (1.8–3.0)	2.2 (1.8–2.8)	< 0.001	2.3 (1.7–3.4)	2.0 (1.5–2.8)	< 0.001
DF	1088	607	481	68 (60–74)	66 (59–73)	< 0.001	71 (65–76)	70 (63–76)	< 0.001	62 (56–70)	61 (55–67)	0.12
BF	1298	695	603	0.7 (0–3.0)	0.7 (0–2.9)	0.41	1.0 (0–3.9)	1.1 (0.1–3.7)	0.67	0.4 (0–2.1)	0.3 (0–2.0)	0.39

Data reported as median (25th percentile-75th percentile)

*BF* Backflow, *DF* Diastolic fraction, *MGF* Mean graft flow, *PI* Pulsatility index, *ONCAB* On-pump coronary artery bypass, *OPCAB* Off-pump coronary artery bypass

There were statistically significant differences in pre- vs. post-protamine MAPs in the 768 grafts (with the MAP generally higher post-protamine. See Additional file 1 for detailed MAP table).

Secondary analysis, including only grafts with pre- and post-protamine ACI above 30% and MAP within the same range pre- and post-protamine, showed that MGF increased post-protamine for venous grafts from 30 to 32 mL/min (*P* <  0.001), but remained unchanged for arterial grafts (28 mL/min, *P* = 0.44). Concerning the single grafts with the same MAP range pre and post protamine, the 95th percentile for MGF exceeded 25 mL/min. No correlation was found between the outliers (i.e., grafts with a reduction of more than 25 mL/min in flow) and graft or anastomosis revision (as defined in the original REQUEST trial, Table 1 [8]).

**Fig. 2:**
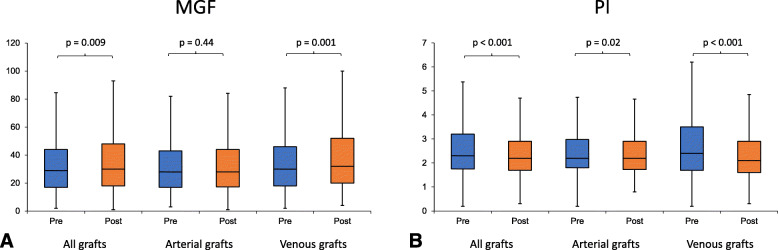
Mean graft flows (**A**) and pulsatility indices (**B**) pre- and post-protamine: single grafts with pre- and post-protamine TTFM with ACI > 30 (both pre- and post-protamine) and same MAP range (for each graft). Data reported as medians. Boxes represent the 1st to 3rd (IQR) quartile while the whiskers are from the min to the max values and are truncated to no longer than 1.5 times the IQR. ACI = acoustic coupling index; MAP = mean arterial pressure; MGF = mean graft flow; PI = pulsatility index; TTFM = transit-time flowmetry

PI decreased in venous (2.4 to 2.1, *P* <  0.001), and was numerically unchanged, but statistically significant for arterial grafts (2.2, *p* = 0.02). DF decreased post-protamine for arterial grafts (71% pre-protamine vs 70% pre-protamine, *p* <  0.001) but was unchanged for venous grafts (61% pre-protamine vs 60% post-protamine, *p* = 0.18). Finally, BF was unchanged for both arterial (0.9% vs 1.1%, *P* = 0.78) and venous grafts (0.4% vs. 0.5%, *p* = 0.96) (Table 3 and Fig. 2).

**Table 3:** Transit-time flowmetry parameters before and after administration of protamine: single grafts with pre- and post-protamine TTFM with ACI > 30 (both pre- and post-protamine) and same MAP range (for each graft)

	N all grafts	N arterial grafts	N venous grafts	All			Arterial grafts			Venous grafts		
				Pre-protamine	Post-protamine	*p* value	Pre-protamine	Post-protamine	*p* value	Pre-protamine	Post-protamine	*p* value
MGF	559	318	241	29 (17–44)	30 (18–48)	0.009	28 (17–43)	28 (17–44)	0.44	30 (18–46)	32 (20–52)	0.001
PI	559	318	241	2.3 (1.8–3.2)	2.2 (1.7–2.9)	< 0.001	2.2 (1.8–3.0)	2.2 (1.7–2.9)	0.02	2.4 (1.7–3.5)	2.1 (1.6–2.9)	< 0.001
DF	489	289	200	68 (60–73)	66 (58–73)	< 0.001	71 (65–76)	70 (63–75)	< 0.001	61 (56–69)	60 (54–66)	0.18
BF	552	315	237	0.8 (0–3.0)	0.9 (0–3.0)	0.81	0.9 (0–3.9)	1.1 (0.2–3.7)	0.78	0.4 (0–2.3)	0.5 (0–2.2)	0.96

Data reported as median (25th percentile-75th percentile)

*BF* Backflow, *DF* Diastolic fraction, *MAP* Mean arterial pressure, *MGF* Mean graft flow, *PI* Pulsatility index, *ONCAB* On-pump coronary artery bypass, *OPCAB* Off-pump coronary artery bypass

## Discussion

In this novel study comparing pre- and post-protamine measurements, we found that MGF was unchanged for arterial but slightly increased for venous grafts (30 mL/min vs. 32 mL/min, respectively, *P* <  0.001) after protamine administration, once MAP had been corrected for. The 95th percentile for MGF was 25 mL/min (i.e., in 95% of all single grafts after accounting for MAP, the MGF increased, stayed the same, or decreased in less than 25 mL/min). PI slightly decreased (especially for venous grafts. Of note, though the PI of the arterial grafts did not change, this finding was statistically significant since the distribution of the difference of pre- and post-protamine PI had a tendency towards negative values representing higher values pre-protamine compared to post-protamine. However, these differences were mostly symmetrically distributed around zero (see figure 1 in the Additional file 1). These differences were, in our opinion, not clinically significant.

TTFM can provide detailed information regarding graft flow and patency; however, proper handling technique is paramount. Graft patency and flow are assessed by four variables: MGF, PI, BF%, and DF% [9]. Each variable adds its own unique piece of information, and no single variable can be taken in isolation during decision making for graft revision.

Despite its clinical utility, TTFM has not yet achieved widespread acceptance. Part of this can be explained by the small size of the cohorts and studies used to research TTFM and their differing cutoff values for predicting graft failure. The ESC/EACTS guidelines include a recommendation for TTFM graft assessment (class of recommendation IIa, level of evidence B) [7] with cutoff values for MGF of > 20 mL/min and a PI < 5 being the most widely cited values [6, 10].

The diagnostic accuracy of TTFM is still a matter of debate with different values of sensitivity and specificity reported [6, 11]. Furthermore, the lack of universally accepted cut off values results in divergent values used in different studies.

Using a patency prediction model based on virtual machine learning, Mao et al. have recently demonstrated that the implementation of TTFM parameters with clinical and hemodynamic characteristics significantly increased its sensitivity and specificity values [12]. Though this has not yet been clinically applied, it appears that the addition of high frequency ultrasound (HFUS) increases the sensitivity and specificity to detect a problematic graft dramatically [13].

The recently published REQUEST trial [8] was the first prospective, multicenter trial to examine the influence of intraoperative graft assessment with TTFM and/or HFUS on decision-making in multivessel CABG. In the trial, changes in surgical strategy were performed in 25.2% of patients, with most of these based only on an abnormal TTFM or HFUS. These changes included a revision of 7.8% of completed grafts. Very low rates of peri-operative adverse events, particularly mortality, were observed. Of note, 37.5% of patients in the trial were above the age of 70, which is reflective of current real-world practice. Previous reports have shown that TTFM parameters in the elderly population is similar to that of the younger population [14].

Mean arterial pressure (MAP) has been known to effect MGF. Except at the extremes of MAP, MGF is directly proportional to MAP when coronary vascular resistance is unaltered. For instance, Balacumaraswami et al. [15], when reporting on TTFM parameters in on- vs. off-pump CABG, devised the flow/pressure ratio which was calculated as a ratio of MGF-to-MAP in order to account for the effects of MAP on graft flow. In order to account for the effect of MAP, after our first analysis of TTFM parameters (702 patients with a total of 1335 grafts) we conducted a secondary analysis of TTFM parameters only in the 368 patients with 567 grafts that had the same MAP reported before and after the administration of protamine.

As previously mentioned, despite TTFM’s potential to provide valuable data in coronary surgery (especially when combined with HFUS), it is underutilized. Using a retrospective analysis of the REQUEST trial, we aimed to add another parameter to TTFM and to improve TTFM’s sensitivity and specificity. Based on clinical observations of a change in TTFM parameters after the administration of protamine, we retrospectively analyzed the REQUEST trial to quantify this change in parameters. To reduce the effects of MAP on graft flow we compared the various TTFM parameters using only single grafts with similar MAP range before and after the administration of protamine. Sequential grafts were excluded to minimize confounding, since the effect of protamine on single grafts had not yet been quantified. We found no reduction in flow in the post-protamine measurements (there was even a small, albeit clinically insignificant, rise in flow in venous grafts and arterial grafts). There was also some improvement in the PI post–protamine (mainly in venous grafts but not in arterial grafts). DF decreased post-protamine for arterial grafts but was unchanged for venous grafts and BF was unchanged for both graft types. Of note, reports on flows in arterial vs. venous conduits usually report higher flows in venous conduits [16], but this is not always true when comparing the radial artery to venous grafts [17]. We did not differentiate between radial grafts and other arterial grafts which might have yielded slightly different results.

Protamine causes a well-known series of transient effects during reversal of heparin anticoagulation, including systemic hypotension, pulmonary hypertension, bradycardia and bronchoconstriction [18]. Belboul et al. have also investigated the effects of protamine on the epicardial microflow [19]. They demonstrated a dynamic effect: after transient vasodilation (resulting in an improvement of MGF), vasospasm was detected (thus making vascular resistance higher and decreasing mean flow locally). Protamine has been demonstrated to induce vasoconstriction by inducing leucocyte degranulation [20] and to alter the viscosity of blood by activating platelet adherence to intact endothelium of arterioles thereby inducing thrombus formation [21]. Of note, a recent publication from Korea did not show any association between blood viscosity and TTFM [22]. However, these (mostly) old data have neither been subsequently confirmed in vivo nor been linked to TTFM values after protamine administration in CABG.

### Limitations

This study was a retrospective review of the data from the original REQUEST trial that was not designed to answer the question of how TTFM parameters change after protamine administration. Use of TTFM requires committed training both for the application of the device and the interpretation of the data. Only in-hospital outcomes of patients were tracked so no long-term clinical outcomes or angiographic outcomes are available to correlate with our findings. We did not examine the different effects of protamine administration on graft flow by coronary territory, in preticular compering the left anterior descending to non-left anterior descending targets. Finally, only single grafts were analyzed because we wanted to reduce any additional confounding with the inclusion of sequential grafts, yet sequential grafts are routinely performed in clinical practice.

## Conclusions

We found no clinically significant difference in TTFM parameters before and after the administration of protamine. Furthermore, even when examining outliers (i.e., grafts with a reduction of MGF > 25 mL/min with protamine administration), we did not find a higher rate of graft revision. This finding might obviate the need for performing TTFM before and after protamine administration since a single TTFM measurement may suffice to achieve reliable data on each graft’s performance. Depending on the specific clinical situation and intraoperative changes, more measurements may be informative (i.e., with changes in MAP, volume status of the patient, and more).

## Supplementary Information

**Additional file 1.**

## Data Availability

The datasets used and/or analyzed during the current study are available from the corresponding author on reasonable request.

## Abbreviations

CABG: Coronary Artery Bypass Graft
TTFM: Transit-Time Flow Measurement
MGF: Mean Graft Flow
PI: Pulsatility Index
DF: Diastolic Filling
BF: Backflow
MAP: Mean Arterial Pressure
HFUS: High Frequency Ultrasound
ONCAB: On-Pump Coronary Artery Bypass
OPCAB: Off-Pump Coronary Artery Bypass
ACI: Acoustic Coupling Index
