# Supplementary material for: Transit time flow measurement of coronary bypass grafts before and after protamine administration
Source: J Cardiothorac Surg. 2021 Jul 9;16:195. doi: 10.1186/s13019-021-01575-y (PMC8268198; doi:10.1186/s13019-021-01575-y)
Supplement: Supplementary file 1 — Additional file 1. [file 13019_2021_1575_MOESM1_ESM.docx]

Appendix:

Figure S1:

**Table S1**: Mean arterial pressure before and after administration of protamine: single grafts with pre- and post-protamine TTFM with ACI > 30 (both pre- and post-protamine).

|  |  | N all grafts | N arterial grafts | N venous grafts | All | | | Arterial grafts | | | Venous grafts | | |
| --- | --- | --- | --- | --- | --- | --- | --- | --- | --- | --- | --- | --- | --- |
|  |  |  |  |  | Pre-protamine | Post-protamine | p value | Pre-protamine | Post-protamine | p value | Pre-protamine | Post-protamine | p value |
| All | MGF | 1319 | 704 | 615 | 69 (69-80) | 80 (69-80) | < 0.001 | 69 (69-80) | 80 (69-80) | 0.06 | 69 (58-80) | 80 (69-80) | < 0.001 |
|  | PI | 1319 | 704 | 615 | 69 (69-80) | 80 (69-80) | < 0.001 | 69 (69-80) | 80 (69-80) | 0.06 | 69 (58-80) | 80 (69-80) | < 0.001 |
|  | DF | 1088 | 607 | 481 | 69 (69-80) | 80 (69-80) | < 0.001 | 69 (69-80) | 80 (69-80) | 0.14 | 69 (58-80) | 80 (69-80) | < 0.001 |
|  | BF | 1298 | 695 | 603 | 69 (69-80) | 80 (69-80) | < 0.001 | 69 (69-80) | 80 (69-80) | 0.07 | 69 (58-80) | 80 (69-80) | < 0.001 |
| ONCAB | MGF | 799 | 390 | 409 | 69 (58-80) | 69 (58-80) | < 0.001 | 69 (58-80) | 69 (58-80) | 0.02 | 69 (58-80) | 69 (69-80) | 0.007 |
|  | PI | 799 | 390 | 409 | 69 (58-80) | 69 (58-80) | < 0.001 | 69 (58-80) | 69 (58-80) | 0.02 | 69 (58-80) | 69 (69-80) | 0.007 |
|  | DF | 625 | 327 | 298 | 69 (58-80) | 69 (69-80) | < 0.001 | 69 (58-69) | 69 (58-80) | 0.02 | 69 (58-80) | 69 (69-80) | < 0.001 |
|  | BF | 790 | 387 | 403 | 69 (58-80) | 69 (58-80) | < 0.001 | 69 (58-80) | 69 (58-80) | 0.03 | 69 (58-80) | 69 (69-80) | 0.01 |
| OPCAB | MGF | 520 | 314 | 206 | 80 (69-80) | 80 (69-80) | 0.03 | 80 (69-80) | 80 (69-80) | 0.86 | 80 (69-80) | 80 (69-80) | 0.001 |
|  | PI | 520 | 314 | 206 | 80 (69-80) | 80 (69-80) | 0.03 | 80 (69-80) | 80 (69-80) | 0.86 | 80 (69-80) | 80 (69-80) | 0.001 |
|  | DF | 463 | 280 | 183 | 80 (69-80) | 80 (69-80) | 0.07 | 80 (69-80) | 80 (69-80) | 0.71 | 80 (69-80) | 80 (69-80) | 0.001 |
|  | BF | 508 | 308 | 200 | 80 (69-80) | 80 (69-80) | 0.02 | 80 (69-80) | 80 (69-80) | 0.82 | 80 (69-80) | 80 (69-80) | < 0.001 |
| Post-protamine MAP higher | | |  |  |  |  |  |  |  |  |  |  |  |

Data reported as median (25^th^ percentile-75^th^ percentile).

BF = backflow; DF = diastolic fraction; MAP = mean arterial pressure: MGF = mean graft flow; PI = pulsatility index; ONCAB = on-pump coronary artery bypass; OPCAB = off-pump coronary artery bypass
